# Factors Associated With Anxiety Symptoms in Older Adults Attending an Outpatient Geriatric Service: A Cross‐Sectional Study

**DOI:** 10.1002/agm2.70018

**Published:** 2025-04-18

**Authors:** Gianella Grajeda‐León, Victoria Azurin‐Gonzales, Zulema Mamani‐Condori, Alvaro M. Ñaña‐Cordova, Marina A. Bustamante‐Ordoñez, Fiorella Neyra‐Cordova, José F. Parodi, Fernando M. Runzer Colmenares

**Affiliations:** ^1^ Carrera de Medicina Humana Universidad Científica del Sur Lima Peru; ^2^ Sociedad Cientifica de Estudiantes de Medicina de la Universidad Científica del Sur Lima Peru; ^3^ CHANGE Research Working Group Carrera de Medicina Humana, Universidad Científica del Sur Lima Peru; ^4^ Universidad de San Martín de Porres Facultad de Medicina Humana, Centro de Investigación del Envejecimiento (CIEN) Lima Peru

**Keywords:** anxiety, elderly, Hamilton anxiety rating scale, mental health

## Abstract

**Objective:**

To identify the factors contributing to anxiety among adults aged 65 and older receiving care at the Geriatric Service of the Naval Medical Center.

**Methods:**

An analytical study was conducted through secondary analysis of a database from a study carried out between 2010 and 2015 at the Naval Medical Center of Peru (CEMENA). The data from 1686 participants were analyzed, with anxiety as the dependent variable, measured using the Hamilton Anxiety Rating Scale with a cutoff point of 14. The analysis was performed using STATA software. Bivariate analysis was conducted using the chi‐squared and Student *t*‐tests, while Poisson regression was employed for multivariate analysis to determine the frequencies and associations between anxiety and the various variables.

**Results:**

In the multivariate analysis, anxiety was found to be significantly associated with gait speed (PR 1.11; 95% CI 1.03–1.20), depressive symptoms (PR 1.97; 95% CI 1.81–2.16), polypharmacy (PR 1.14; 95% CI 1.04–1.06), and education level (PR 0.83; 95% CI 0.76–0.90). Additionally, marital status was found to be associated with the likelihood of anxiety: being married (PR 0.64; 95% CI 0.53–0.77), widowed (PR 0.54; 95% CI 0.44–0.66), or divorced (PR 0.63; 95% CI 0.49–0.83) were all linked to a lower probability of anxiety compared to being single.

**Conclusion:**

Factors such as depressive symptoms, polypharmacy, slow gait speed, education level, and marital status were found to be associated with anxiety in older adults.

## Graphical abstract

An analytical study was carried out by secondary analysis of a database of a study performed between 2010 and 2015 at the Naval Medical Center of Peru (CEMENA). the analysis revealed that depressive symptoms, polypharmacy, slow gait speed, education, and marital status were significant contributors to anxiety in this population.

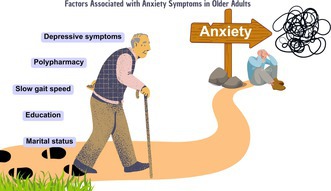

## 1 Introduction

Anxiety is an emotional response triggered by an undesired situation, leading to a series of reactions in cognitive, physiological, and behavioral domains. However, when this emotion escalates into an anxiety disorder, it manifests as fear, excessive worry, anticipatory anxiety, and avoidance behaviors [1]. While this condition can affect individuals of all ages, its prevalence, along with other mental health issues, is particularly high among older adults in certain hospital settings [2].

According to data from the World Health Organization (WHO), by 2050, the proportion of the global population aged 60 years and older is expected to reach 22% [2]. This demographic shift will lead to a higher number of individuals classified as elderly. Among this population, anxiety is the second most prevalent mental disorder, representing a significant clinical challenge with a global incidence of 3.8%, as reported by the WHO [2].

In Peru, one in five individuals experience symptoms of a mental disorder, with anxiety and depression being the most common [3]. These conditions are associated with a significant level of disability [4]. In this context, coping becomes a crucial factor, referring to the cognitive and behavioral efforts made to manage overwhelming situations [5]. For older adults, coping is even more essential, as the aging process leads to a decline in both material and personal psychosocial resources [1]. This reduction in resources makes the elderly more vulnerable to various mental disorders, including anxiety. This underscores the need to equip older adults with a broader set of coping strategies [6] to effectively manage emotionally and physically distressing situations, which can otherwise lead to excessive worry, anxiety episodes, and an undue increase in the use of healthcare services [7].

Anxiety in older adults is influenced by multiple factors. Recent studies have shown that gait speed in the elderly not only reflects physical aging but may also influence changes in neural movement and emotion, potentially increasing the risk of anxiety and depression, which may be linked to dopaminergic dysfunction. However, other factors, such as muscle strength and dysfunction, still require further investigation. Therefore, our study aims to analyze these aspects to provide a more comprehensive understanding [8]. Furthermore, polypharmacy is a significant barrier to the treatment of anxiety disorders in older adults, a population in which the prevalence of anxiety disorders can be as high as 75%. This increases the risk of potentially inappropriate medication use (PIM), with PIM affecting up to 66.6% of these patients. The most commonly used medications in this group include NSAIDs, gastrointestinal drugs, and certain antidepressants [9].

For primary prevention of anxiety, it is important to recognize the factors associated with anxiety in older adults [10]. It is essential to know the context within which this pathology develops or is enhanced [11]. Stressors such as the presence of certain diseases and pandemics have been shown to be related to an increase in anxiety [11, 12]. Other factors related to the individual's social, family, and personal development may also predispose to susceptibility to anxiety [13]. Education is key to older adults' mental health, with higher levels linked to a lower risk of anxiety, likely due to better financial resources, social networks, and coping skills. However, this relationship is not uniform, following a dose–response pattern and stabilizing at high education levels, possibly influenced by factors like “overeducated realism” and sociodemographic differences [14].

Marital status influences older adults' mental health, with marriage linked to better quality of life and lower anxiety. Widowhood and divorce increase vulnerability and loneliness. While some singles report higher psychological satisfaction, more research is needed on emotional connection and social support's impact on anxiety [15].

The presence of anxiety in older adults is associated with conditions that may worsen their overall health [16]. It has also been linked to memory loss and difficulties with executive function, such as organization and planning [17]. Additionally, many older adults have poor awareness of the condition, and even when it is recognized, they often refuse treatment [2]. These factors, along with any comorbidities they may have, contribute to treatment resistance and an increased risk of suicide [18].

There is limited recent information on anxiety disorders, particularly in older adults [19]. Therefore, our study aimed to identify the factors associated with anxiety in older adults at the Naval Medical Center in Peru. Gaining more insight into this issue would provide a foundation for the development of evidence‐based public health policies [20].

## 2 Methods

### 2.1 Study Design and Population

The design of this study was analytical and observational, utilizing the database from the study “Prevalence and Factors Associated with Frailty Among Peruvian Older Adults.” The objective of that study was to identify the prevalence and factors associated with frailty in older adults selected through nonprobability sampling [21].

The original study had the following inclusion criteria: Individuals aged 60 years and older seen in an outpatient clinic. The exclusion criteria included severe functional impairments, inability to walk, refusal to provide informed consent, HIV/AIDS diagnosis, cancer diagnosis, hospitalization, or being under home care at the time of recruitment. These were all the exclusion criteria in the original study.

Additionally, for the present secondary analysis, we included data from the original study participants, excluding those with abnormal results on the Mini Mental State Examination (Peruvian adaptation). The cutoff points for this test were 27 for individuals with more than 7 years of education, 23 for those with 4–7 years, 21 for those with 1–3 years, and 18 for illiterate individuals [22]. Participants with missing data on the Hamilton Anxiety Scale were also excluded. The final sample consisted of data from 1686 participants. To calculate statistical power, we referenced a study that reported an anxiety prevalence of 49.5% [23]. Based on our sample size and a confidence level of 5%, a power of 99.9% was calculated using Open Epi 3.0 software.

### 2.2 Definition of Variables

Anxiety symptoms: Assessed with the Hamilton Anxiety Scale validated in Spanish [20] which consists of a questionnaire of 14 questions, each scored on a scale from 0 to 4 points, distinguishing the different degrees of anxiety, where 0 = absence/no symptoms; 1 = mild/occurs irregularly; 2 = moderate/presentation of symptoms over a longer period; 3 = severe/presentation is continuous and dominates the subject's life; and 4 = very severe/being incapacitating. This scale assesses both psychic and somatic anxiety symptoms, with the score ranging from 0 to 54, and a score < 14 was considered absence of anxiety [21]. The questionnaire was taken during the geriatric consultation by professionals trained in research, in a private setting.

Anxiety Symptoms: Anxiety was assessed using the Hamilton Anxiety Scale, validated in Spanish [24]. This scale consists of 14 questions, each scored on a scale from 0 to 4, to distinguish varying degrees of anxiety. The scoring is as follows: 0 = absence/no symptoms; 1 = mild/occurs irregularly; 2 = moderate/presentation of symptoms over a longer period; 3 = severe/presentation is continuous and dominates the subject's life; and 4 = very severe/being incapacitating. The scale evaluates both psychic and somatic anxiety symptoms, with a total score ranging from 0 to 54. A score of < 14 was considered indicative of the absence of anxiety [25]. The questionnaire was administered during the geriatric consultation by trained professionals.

Sociodemographic Variables: Age (in years), sex (female or male), marital status (single, married, widowed, divorced/separated), education level (≥ 11 years or < 11 years), and self‐reported living situation (living alone: yes or no).

Polypharmacy: five drugs per day (yes or no) [21].

Falls: At least one fall in the last year (yes or no) [21].

Comorbidities assessed included diabetes mellitus, chronic kidney disease, hypertension, periodontal disease, chronic obstructive pulmonary disease, obesity (BMI > 30 kg/m^2^), vascular insufficiency, congestive heart failure, urinary incontinence (using one item from the Edmonton scale) [26], hip fractures, other fractures, hypothyroidism, stroke, and knee osteoarthritis. Data were collected from medical histories, except for BMI and urinary incontinence, which were assessed during the study consultation.

Functional impairment was assessed using the Barthel Index, which evaluates basic activities of daily living. The score ranges from 0 to 100, with a cutoff point of < 95 considered indicative of impaired functionality [27].

Muscle Strength: Muscle strength was quantitatively assessed using an electronic dynamometer, measuring the pressure exerted by the dominant hand in kilograms (kg). A cutoff point of < 27 kg for men and < 16 kg for women was used to determine impaired muscle strength [28].

Gait speed was measured in meters per second (m/s) by quantifying the time it takes to walk 4 m. A cutoff point of 0.8 m/s was used, with a result below this threshold considered indicative of impaired gait [29].

Depressive symptoms were assessed using the 5‐item Yesavage questionnaire. A score of 3 or more (based on “yes” or “no” responses) was considered indicative of depression [27].

### 2.3 Statistical Analysis

Statistical analysis of data from 1686 participants was performed using STATA 18.0 for Windows. Bivariate analysis was conducted using Student's *t*‐tests and chi‐squared tests (chi square), with a *p* value of < 0.05 considered statistically significant, and a 95% confidence interval (CI) applied in all cases. Additionally, the frequency of anxiety was determined. For the multivariate analysis, Poisson regression was used to calculate crude and adjusted prevalence ratios (PR) to assess the association between anxiety and various covariates. The final model included variables that showed a significant association in the bivariate analysis. Poisson regression was selected because the goal was to model count data, particularly in studies analyzing the frequency of events (such as anxiety in this case). This method is well suited for analyzing rates or counts of events in populations, providing accurate estimates of the association between anxiety and the covariates. Multicollinearity was assessed using the VIF (Variance Inflation Factor) command. Poisson regression was chosen because cross‐sectional studies use the calculation of prevalence ratios instead of odds ratios (OR), which generally leads to less overestimated results.

## 3 Results

A total of 1686 participants were included, with a mean age of 78.3 years (standard deviation [SD] ± 8.5). Males represented 59.8% (*n* = 1000) of the sample. Regarding educational level, 73.2% (*n* = 1200) had ≥ 11 years of education. In terms of marital status, the majority were married (73.8%; *n* = 1199), followed by widowed individuals (19.2%; *n* = 312). Additionally, 73.2% (*n* = 1450) reported not living alone. Concerning comorbidities, 74.2% (*n* = 1251) had ≥ 2 chronic conditions. Functional impairment was identified in 64.9% (*n* = 1092) of participants, while 34.2% (*n* = 575) exhibited weak muscle strength. Impaired walking speed was observed in 30.7% (*n* = 496) of participants. Furthermore, 25.5% (*n* = 431) exhibited depressive symptoms, 32.8% (*n* = 548) had polypharmacy, and 59.6% (*n* = 1006) reported falls in the past year. In terms of anxiety, the frequency observed was 59% (*n* = 995) (Table 1).

**TABLE 1:** Characteristics of the study population (*n* = 1686).

Variable	*n*	%
Age (mean ± SD)	78.31	8.56
Gender^a^		
Female	684	40.62
Male	1000	59.38
Education^a^		
< 11 years	438	26.74
≥ 11 years	1200	73.26
Marital status^a^		
Single	49	3.02
Married	1199	73.83
Widower	312	19.21
Separated/Divorced	64	3.94
Living alone^a^		73.27
No	1450	73.28
Yes	232	13.79
Number of comorbidities		
0	95	5.63
1	340	20.17
≥ 2	1251	74.20
Functional impairment^a^		
No	589	35.04
Yes	1092	64.96
Muscle strength^a^		
Normal	1105	65.77
Altered	575	34.23
Walking speed^a^		
Normal	1119	69.29
Altered	496	30.71
Depressive symptoms^a^		
No	1254	74.42
Yes	431	25.58
Polypharmacy^a^		
No	1120	67.15
Yes	548	32.85
Falls		
No	680	40.31
Yes	1006	59.67
Anxiety		
No	691	41.0
Yes	995	59.0

Abbreviation: SD, standard deviation.

^a^ Complete data were not obtained from the database.

The bivariate analysis revealed several significant associations. Age was one such factor, with individuals experiencing anxiety having a statistically significant higher mean age of 79 ± 8.55, compared to 77.31 ± 8.48 in those without anxiety. A significant association was also found between anxiety and educational level, with 653 (68.02%) individuals who had more than 11 years of education reporting anxiety. Additionally, a higher prevalence of anxiety was observed in married individuals (*n* = 705; 74.13%), followed by widowed individuals (*n* = 171; 17.98%). Regarding physical health, individuals with anxiety had a higher prevalence of weak muscle strength (*n* = 416; 42.06%) and a significantly slower walking speed (*n* = 351; 37.42%). The analysis also showed that individuals with anxiety had a significantly higher prevalence of depressive symptoms (*n* = 429; 43.16%). Finally, a trend toward polypharmacy was observed among individuals with anxiety (*n* = 466; 47.45%) (Table 2).

**TABLE 2:** Bivariate analysis between study covariates and anxiety (*n* = 1686).

Variables	Without anxiety	Anxiety	*p*
Age (Mean ± SD)	77.31 ± 8.48	79 ± 8.55	< 0.001
Gender			0.300
Female	291 (42.11)	393 (39.58)	
Male	400 (57.89)	600 (60.42)	
Education (*n* %)			< 0.001
≤ 11 years	131 (19.32)	307 (31.98)	
> 11 years	547 (80.68)	653 (68.02)	
Marital status			< 0.001
Single	7 (1.04)	42 (4.42)	
Married	494 (73.4)	705 (74.13)	
Widower	141 (20.95)	171 (17.98)	
Separated/Divorced	31 (4.61)	33 (3.47)	
Living alone (*n* %)			0.045
Yes	580 (84.18)	870 (87.61)	
No	109 (15.82)	123 (12.39)	
No. of comorbidities			0.900
0	41 (5.93)	54 (5.43)	
1	141 (20.41)	199 (20.0)	
≥ 2	509 (73.66)	742 (74.57)	
Functional impairment (*n* %)			0.200
Normal	252 (36.68)	337 (33.9)	
Altered	435 (63.32)	657 (66.1)	
Muscle strength (*n* %)			< 0.001
Normal	532 (76.99)	573 (57.94)	
Altered	159 (01.23)	416 (42.06)	
Walking speed (*n* %)			< 0.001
Normal	532 (78.58)	587 (62.58)	
Altered	145 (21.42)	351 (37.42)	
Depressive symptoms (*n* %)			< 0.001
No	689 (99.71)	565 (56.84)	
Yes	2 (0.29)	429 (43.16)	
Polypharmacy (*n* %)			< 0.001
No	604 (88.05)	516 (52.55)	
Yes	82 (11.95)	466 (47.45)	
Falls			0.204
No	292 (42.26)	388 (38.99)	
Yes	399 (57.74)	607 (61.01)	

*Note*: For the analysis of the numerical variables, the Student *t*‐test was used. For the analysis of categorical variables, the chi‐squared test was used.

Abbreviation: SD, standard deviation.

In the regression analysis, age and weak muscle strength were associated with anxiety in the crude model, but these associations were not significant in the adjusted model. In terms of educational level, incomplete or complete school education was considered a protective factor against anxiety compared with technical or higher education (PR 0.83; 95% CI 0.76–0.90). Similarly, being married, widowed, or divorced was found to be protective against anxiety when compared to being single. These variables (education and marital status) remained significant in the adjusted model. Additionally, depressive symptoms (PR 1.97; 95% CI 1.81–2.16), polypharmacy (PR 1.14; 95% CI 1.04–1.06), and slow walking speed (PR 1.11; 95% CI 1.03–1.2) were shown to be consistently associated with anxiety in both models (Table 3).

**TABLE 3:** Poisson regression to determine factors associated with anxiety in the study sample (*n* = 1686).

Variables	Crude model: PR (95% CI)^a^	Adjusted model: PR (95% CI)^b^
Age in years	1.010 (1.010–1.020)	1.010 (0.990–1.010)
Education		
Technical/Higher	Reference	Reference
Incomplete/Complete school	0.780 (0.720–0.840)	0.830 (0.760–0.900)
Live alone		
No	Reference	Reference
Yes	0.880 (0.780–1.000)	0.970 (0.870–1.090)
Civil status		
Single	Reference	Reference
Married	0.690 (0.610–0.780)	0.640 (0.530–0.770)
Widower	0.640 (0.55–0.740)	0.540 (0.440–0.660)
Separated/Divorced	0.600 (0.46–0.780)	0.630 (0.490–0.830)
Weak muscle strength		
No	Reference	Reference
Yes	1.400 (1.290–1.500)	1.040 (0.960–1.120)
Slow walking speed		
No	Reference	Reference
Yes	1.350 (1.250–1.460)	1.11 (1.03–1.20)
Polypharmacy		
No	Reference	Reference
Yes	1.850 (1.720–1.980)	1.140 (1.040–1.260)
Depression		
No	Reference	Reference
Yes	2.210 (2.010–2.250)	1.970 (1.810–2.160)

Abbreviations: CI, confidence interval; PR, prevalence ratio.

^a^ Prevalence ratios and 95% confidence interval.

^b^ Model adjusted for age in years, education, living alone, marital status, muscle strength, walking speed, polypharmacy, and depression.

## 4 Discussion

In our study, the frequency of anxiety was found to be 59%, which is notably higher compared to other studies, where the prevalence of anxiety ranged from 8% to 49.9% [23, 30, 31, 32]. It is important to note that the latter study was conducted in a psychiatric hospital [32]. The differences in prevalence across these studies are primarily attributed to variations in sample characteristics and the tools used to assess anxiety [30]. In our sample, the high frequency of anxiety may be due to most participants being Peruvian military personnel and their families, who were more directly involved in armed conflicts than the general population. This closer connection, particularly to the terrorism events of 1980–1992, may have contributed to the observed mental health issues. Historical accounts suggest that many citizens, especially men, were forced into military service during this period, which has been linked to long‐term mental health consequences, including anxiety and depression [33].

Most studies suggest that higher educational attainment is linked to a lower likelihood of experiencing anxiety, as individuals with more education typically have better resources to manage the disorder and access treatment [23, 30]. However, our study showed a different trend: Higher education was linked to a higher prevalence of anxiety. This may be due to factors like self‐imposed pressure, work‐related stress, and increased symptom awareness. Other research suggests that higher education often involves greater academic and occupational demands, as well as higher self‐expectations, which may explain our findings [34].

In our sample, both men and women experienced anxiety, but it was more prevalent in men, likely due to the predominance of retired military personnel and their families. This contrasts with other studies that typically show a stronger anxiety association in women. One possible explanation for this discrepancy is that men may be less likely to report anxiety symptoms due to fears of judgment or stigmatization [32].

Cabanas‐Sánchez et al. found a significant relationship between muscle strength and the risk of depression and anxiety in adults without major mental health disorders. In contrast, our study found that older adults with anxiety had higher muscle strength (42.06%) but slower walking speed (37.42%), potentially due to fear of falling. These results suggest that maintaining muscle strength may protect against anxiety and depression. A 5 kg decrease in grip strength increased the risk of depression by 13% and anxiety by 12% [33]. Further research is needed to explore this relationship and its clinical implications.

A study has shown that slow walking speed is associated with anxiety, noting alterations in walking cadence, shorter stride length, and decreased balance when standing on one foot, all of which contribute to longer task completion times [29]. Similarly, older adults with slow walking speed and cardiovascular disease were found to experience greater anxiety, likely due to fear of falling [35]. In our study, we suggest that anxiety in older adults may be linked to a higher disease burden, frailty, and functional decline, which can worsen psychological distress and reduce quality of life. This aligns with findings that older adults with anxiety face a 40% higher risk of mobility limitations, such as difficulty walking and climbing stairs [36].

We found that the most significant factor associated with anxiety was the presence of depression symptoms. This finding aligns with other studies, where the co‐occurrence of anxiety and depression ranges from 14% to 49% [23, 32]. Both disorders share similar biological mechanisms, which can lead to their coexistence as a mixed anxiety‐depressive disorder. This is particularly common in older adult populations [18, 32]. Additionally, anxiety is often an occult comorbidity in various psychiatric disorders in older adults, with depression being one of the most frequent conditions associated with it [32]. Both mental disorders can cause significant long‐term functional impairment, and when they coexist, the likelihood of anxiety developing increases [32]. Therefore, it is crucial to screen for anxiety when diagnosing either of these disorders.

Polypharmacy was also strongly associated with anxiety in our study, consistent with findings from a Thai study linking anxiety to high drug use. Individuals with polypharmacy often have more chronic conditions and experience increased anxiety, such as fear of death [37]. This relationship could be bidirectional, as polypharmacy may coexist with anxiety, exacerbating symptoms like chest pain, gastrointestinal issues, and musculoskeletal disorders. These symptoms often require increased medication use for management [38].

The present study has several limitations. First, the sample was limited to retired military personnel and their families from a military hospital, which may affect the generalizability of the results to the broader elderly population in Peru. Additionally, the cross‐sectional design of the study prevents the establishment of causal relationships between the main variables. Furthermore, anxiety was assessed using a screening scale rather than the Diagnostic and Statistical Manual of Mental Disorders, Fifth Edition (DSM‐V), which could lead to either over‐ or under‐diagnosis. Future studies should adopt cohort designs to explore potential associations with greater precision and use the DSM‐V for a more accurate diagnosis of anxiety. Finally, to validate the results, we recommend conducting multicenter studies with more diverse populations.

Despite these limitations, the study is supported by a large database assessed by medical specialists using validated instruments, which strengthens its findings. These results provide valuable insights that contribute to the existing body of knowledge on the subject.

## 5 Conclusions

We have identified several factors that may be associated with anxiety, including depressive symptoms, polypharmacy, slow walking speed, being single, and having more than 11 years of education. Therefore, it is crucial for primary care physicians and healthcare providers working with older adults to be trained in recognizing the factors that can contribute to anxiety, enabling them to detect and address the disorder more effectively. This highlights the importance of adopting a holistic approach that considers these associated factors in older adults, ultimately improving the management of anxiety and preventing its complications.

## Author Contributions

Conceptualization, validation, writing – original draft, Writing – review and editing: G.G.‐L., V.A.‐G., Z.M.‐C., A.M.Ñ.‐C., M.A.B.‐O., F.N.‐C., J.F.P., F.M.R.C. Data curation: G.G.‐L., V.A.‐G., Z.M.‐C., A.M.Ñ.‐C., M.A.B.‐O. Formal analysis: A.M.Ñ.‐C., F.N.‐C., J.F.P., F.M.R.C. Investigation: G.G.‐L., V.A.‐G., Z.M.‐C. Methodology: M.A.B.‐O., F.N.‐C., J.F.P., F.M.R.C. Project administration: A.M.Ñ.‐C., M.A.B.‐O., F.N.‐C., J.F.P., F.M.R.C. Supervision: V.A.‐G., Z.M.‐C.

## Ethics Statement

The present study used a database that does not allow participant identification and received approval for the present secondary analysis from the Ethics Committee of the Universidad Cientifica del Sur (code 706‐2019‐PRE15).

## Conflicts of Interest

The authors declare no conflicts of interest.
